# 2H/4H-Chromenes—A Versatile Biologically Attractive Scaffold

**DOI:** 10.3389/fchem.2020.00623

**Published:** 2020-08-05

**Authors:** Vinit Raj, Jintae Lee

**Affiliations:** School of Chemical Engineering, Yeungnam University, Gyeongsan-si, South Korea

**Keywords:** 2H/4H-chromenes, reaction mechanism, synthetic strategies, biological activities, structure-activity relationship

## Abstract

2H/4H-chromene (2H/4H-ch) is an important class of heterocyclic compounds with versatile biological profiles, a simple structure, and mild adverse effects. Researchers discovered several routes for the synthesis of a variety of 2H/4H-ch analogs that exhibited unusual activities by multiple mechanisms. The direct assessment of activities with the parent 2H/4H-ch derivative enables an orderly analysis of the structure-activity relationship (SAR) among the series. Additionally, 2H/4H-ch have numerous exciting biological activities, such as anticancer, anticonvulsant, antimicrobial, anticholinesterase, antituberculosis, and antidiabetic activities. This review is consequently an endeavor to highlight the diverse synthetic strategies, synthetic mechanism, various biological profiles, and SARs regarding the bioactive heterocycle, 2H/4H-ch. The presented scaffold work compiled in this article will be helpful to the scientific community for designing and developing potent leads of 2H/4H-ch analogs for their promising biological activities.

## Introduction

**Figure 1:**
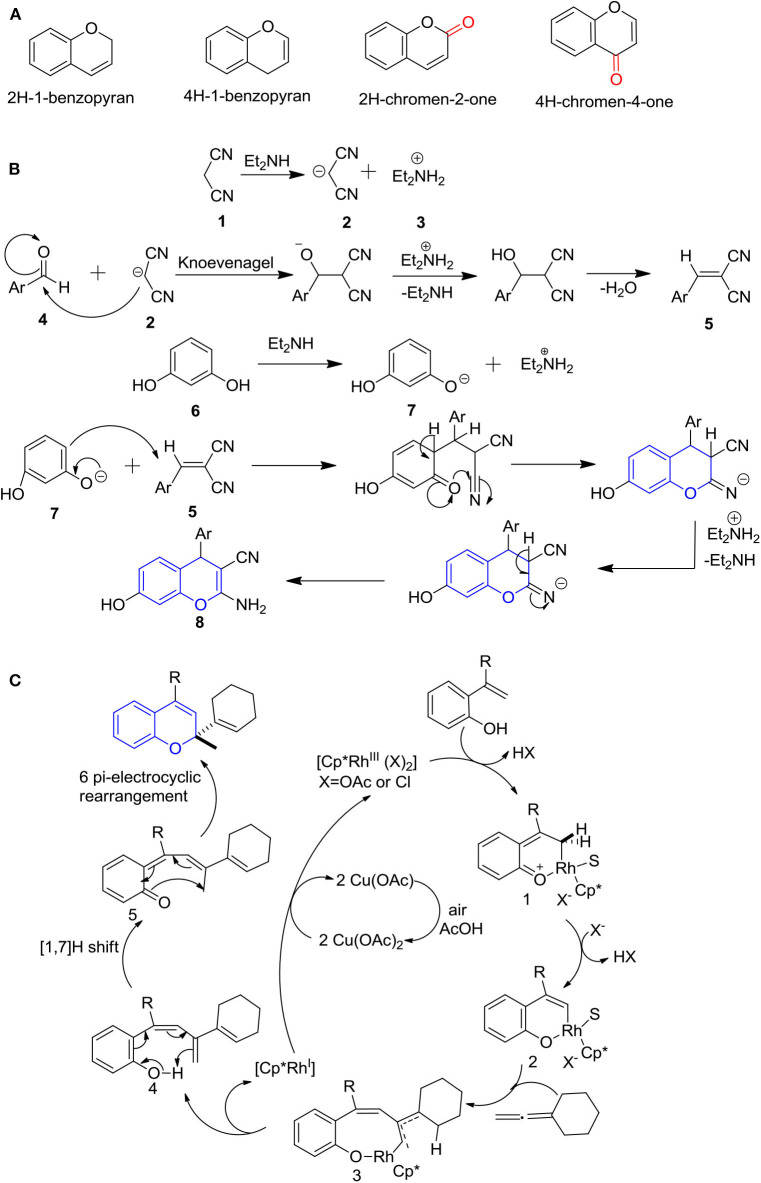
**(A)** Four possible structures of chromenes; **(B)** Plausible reaction mechanisms for the 4H-ch formation; and **(C)** 2H-ch.

Bicyclic oxygen heterocycles-containing a benzene fusion ring at a 5,6-positioned 4H-pyran ring system designated as 4H-chromene (4H-ch) has attracted considerable attention as an important structural motif for the discovery of new drug candidates (El-Gaby et al., 2000). Four common structure motifs are formed, which depend on 9th carbons in the ring, where 8th carbons are sp^2^, and the remaining carbons are sp^3^ hybridized. Importantly, the name of 2H- and 4H-ch depend on the arrangement of sp^3^ carbon associated with the ring oxygen. 4H-chromen-4-one and 2H-chromen-2-one patterns are tracked once sp^3^ carbon is substituted by a carbonyl function, respectively. The name *chromene* is applied to both the 2H- and 4H-form of the molecule, where 4H-ch analogs have been used widely for the decades, as shown in Figure 1A (Goel and Ram, 2009). One of the essential structural features of 4H-ch to impart miscellaneous activity is the occurrence of the fold along the oxygen axis. The molecules containing 2H/4H-ch scaffold exhibit noteworthy potency, such as anticancer (Afifi et al., 2017a; Halawa et al., 2017; Elshaflu et al., 2018; Elnaggar et al., 2019; Luque-Agudo et al., 2019), anticonvulsant (Rawat and Verma, 2016), antimicrobial (Suvarna et al., 2017; Mashhadinezhad et al., 2019), anticholinesterase (Tehrani et al., 2019), antidiabetic activities (Soni et al., 2019), antituberculosis (Zhao et al., 2020), and inhibitory activity against monoamine oxidase (MAO) (Takao et al., 2019).

In addition to the biological potency, medicinal chemists have developed several synthetic methods for the synthesis of 2H/4H-ch derivatives. 4H-ch are generally prepared by a one-pot synthesis using different 2-benzylidene malononitriles and substituted resorcinols in the presence of methanol and calcium hydroxide at room temperature (Kolla and Lee, 2011). In addition, the reported methods consist of the condensation of resorcinol, aryl aldehydes, and malononitrile in the presence of diethylamine under reflux using ethanol as a solvent or 2-aminopyridine as an efficient organo-base-catalyst (Ramesh et al., 2019). Also, 2H-ch analogs are synthesized by the involving Witting-Horner-Emmons and Suzuki-Miyaura cross-coupling pallado-catalyzed reactions (Kaoukabi et al., 2019). Several studies have reported with various methods for the preparation of 2H/4H-ch derivatives, including one-pot synthesis, recyclable catalysts, green methodologies, and reactions in aqueous media to reduce the reaction time, catalyst utilization, and byproducts elimination as well as yield enhancement.

Interestingly, 2H/4H-ch analogs produce the cytotoxic effect in cancer cells, hence, these analogs have been recognized as antitumor agents. Based on these explanations, Anthony et al. (2007) evaluated the underlying mechanisms of 4H-ch-mediated tumor cell cytotoxicity. They reported that 4H-ch analogs generally induce apoptosis *via* interaction through tubulin at binding sites of the colchicine. In this way, they obstruct the polymerization of tubulin, leading to caspase-dependent apoptotic and G2/M cell-cycle arrest in cancer cell death. Therefore, these molecules target and interrupt tumor vasculature (Anthony et al., 2007). Some analogs of 2H/4H-ch triggered the cell apoptosis by the caspse3/7 activation and executioner of DNA fragmentation and also caused a substantial reduction in the cell invasion and cell migration percentage (Alblewi et al., 2019a; Luque-Agudo et al., 2019). Additionally, the potency of substituted-1H-benzimidazol-2-yl fused with 4H-chromen-4-ones analogs was reported, which revealed that 4H-ch analogs were selective inhibitors of formyl peptide receptor-1 (FPR-1), led to blocking the Ca^2+^ flux and inhibited chemotaxis in human neutrophils (Schepetkin et al., 2014). Consequently, with diverse biological potencies, the present structural motifs, 2H/4H-ch, are an excellent core in the preparation of molecules for the treatment of cancer (Anthony et al., 2007). SAR assessments of modifications in these structure frameworks have allowed tailoring of the activity of the 2H/4H-ch nucleus.

The main objective of this review is to compile recent updates of 2H/4H-ch. While there is much literature of 2H/4H-ch analogs for the treatment of cancer, microbial infection, tuberculosis, diabetes, and convulsant, none of the privileged artwork has been reviewed. Therefore, owing to the lack of recent compiled updates about the 2H/4H-ch nucleus and their significance in the development of new compounds with desired biological activities, this article provides a recent updated informative summary of synthetic strategies, reaction mechanisms, biological profiles, and the SAR of 2H/4H-ch. This article is expected to be helpful for medicinal chemists and researchers to design and develop their research work for the discovery of potent lead of 2H/4H-ch to the treatment of life-threatening diseases and other industrial applications.

## Plausible Mechanism for the Synthesis of 2H/4H-Chromenes

While various mechanisms can be employed for the synthesis of the 4H-ch ring, one main and generally used mechanism is ring cyclization by a reaction of substituted resorcinol's and different 2-benzylidene malononitriles or nitrophenylboronic acid as a green catalyst or diethylamide or 1, 8-diazabicyclo[5.4.0]undec-7-ene (DBU) catalyst. Behbahani and Samaei (2014) proposed a plausible synthetic mechanism to the formation of the 4H-ch ring in the occurrence of a multicomponent mixture of a malononitrile, resorcinol, and aromatic aldehyde and diethylamine in ethanol under reflux (Figure 1B). According to the assisted reaction, the formation of cyclic chromene was preceded by the generation of a malononitrile nucleophile along with electrophilic diethylamine. In addition, the carbon of the aromatic aldehyde acts as an electrophile and generates a malononitrile nucleophile that reacts to form a substituted malononitrile. Moreover, resorcinol and diethylamine react together and form the phenoxide anion and electrophilic diethylamine. The generated phenoxide anion of resorcinol and resulting formed substituted malononitrile reacts and cyclizes to form 4H chromenes via an electrophilic substitution reaction (Behbahani and Samaei, 2014; Aminkhani et al., 2019).

The ring cyclization of 2H-ch is initiated by rhodium (III) complex catalysis of a phenolic substrate in the acidic condition (Casanova et al., 2015). In this reaction, rhodium forms a complex with the phenolic substrate as an intermediate complex 1. Further, an arrangement of six-member rhodacycle 2 is proceeded by rearomatization. And the allene incorporates in migratory insertion with rhodacycle 2 to form a pallylic rhodacycle 3. Next, the reductive elimination of pallylic rhodacycle 3 is proceeded by the β-hydride elimination, resulting in the formation of conjugated system 4. Later, dearomatize enone 5 is formed by [1,7]H-shift, which finally gives the 2H-ch by 6π-electrocyclic rearrangement (Figure 1C) (Casanova et al., 2015).

## Synthetic Strategies of 2H/4H-Chromenes

The following synthetic approaches for the synthesis of 2H/4H-ch have been adopted: **(1)** ring cyclization by a reaction of substituted resorcinols and different 2-benzylidene malononitriles, **(2)** ring cyclization *via* nitrophenylboronic acid as a green catalyst and DBU catalyst, **(3)** formation of 2H-ch retinoids hybrid by followed the Suzuki cross-coupling pallado-catalyzed reactions, and **(4)** unexpected [4+2] annulation of alkynyl thioethers with alcohols (o-hydroxybenzyl). The strategies are discussed in the following paragraphs.

### Ring Cyclization of 4H-Chromenes by a Reaction of Substituted Resorcinols and Different 2-Benzylidene Malononitriles

**Figure 2:**
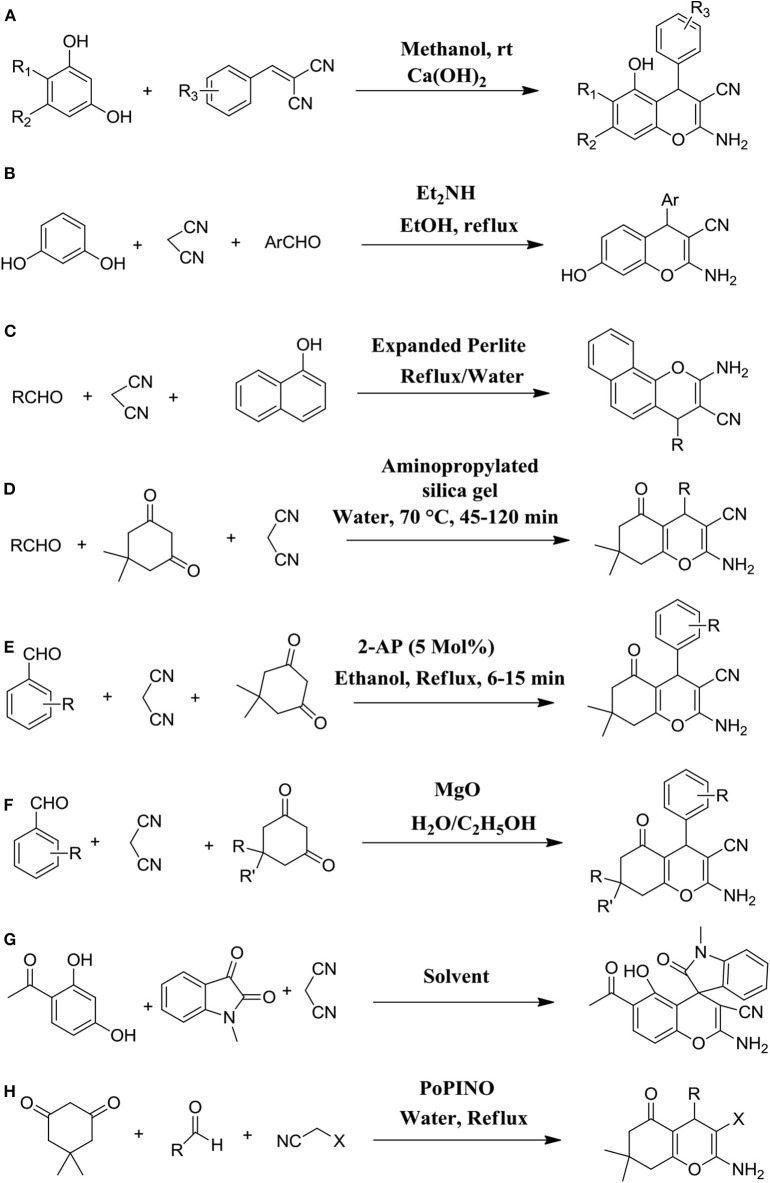
Diverse synthetic strategies of 4H-ch derivatives *via* several catalyzed one-pot reactions as following: **(A)** using resorcinols and 2-benzylidene malononitriles in the presence of Ca(OH)_2_ base; **(B)** by condensation of malononitrile, aryl aldehydes, and resorcinol in the presence of diethylamine; **(C)** by simple mixing of malononitrile, α-naphthol, and aromatic aldehydes in water and catalyzed by expanded Perlite; **(D)** using amino-functionalized silica gel as a base catalyst; **(E)** by using 2-aminopyridine as an organo-base-catalyst; **(F)** by MgO as a base-catalyst; **(G)** using Ca(OH)_2_ base-catalyst; and **(H)** using PoPINO.

New 2-amino-5-hydroxy-4H-ch with numerous additions were synthesized by adopting a one-pot efficient synthetic route using different resorcinols and 2-benzylidene malononitriles in the presence of methanol and calcium hydroxide at room temperature. It was reported that the yield of 4H-substituted-chromenes was very elevated in constant reaction conditions (Figure 2A) (Kolla and Lee, 2011).

Diethylamine has been employed as an efficient organocatalyst in various organic reactions, including the synthesis of chromenes, such as Knoevenagel condensation, aldol condensation, and Michael addition. Hence, Behbahani and Samaei (2014) applied the synthesis of 2-amino-4H-chromenes using a one-pot three-component reaction with utilizing diethylamine. The strategy involved the reflux of resorcinol, aryl aldehydes, and malononitrile with diethylamine and ethanol as a solvent. They reported that the reaction gave excellent yields of products even under mild reaction conditions. In addition, the method was environmentally friendly (Figure 2B) (Behbahani and Samaei, 2014).

Substituted 2-amino-4H-chromenes were reported with tremendous yield and selectivity just by addition aromatic aldehydes, α-naphthol, and malononitrile in water and catalyzed reaction with a green heterogeneous catalyst, called expanded Perlite (Figure 2C). They reported that this procedure had many advantages over the base-catalyzed reaction, including mild reaction conditions, cleaner reaction, a simple work-up procedure, and a higher yield of products. Interestingly, instead of an organic solvent, water was used as a green solvent for these reactions (Abrouki et al., 2013).

### Base Catalyst Recoverable Amino-Functionalized Silica Gel-Based Synthesis of 4H-Chromenes

Inspired by addition reactions such as the Michael, Knoevenagel, and Aldol reactions and base-catalyzed condensation, Joshi et al. (2014) developed an efficient, simple, and environmentally friendly procedure for the preparation of 4H-ch by using recoverable and well-organized silica gel-based amino-functionalized base catalyst. They adopted a reaction of the three-component of cyclic 1,3-diketones, malononitrile, and aldehydes in the water at 70°C. Using this procedure, the 4H-ch were found in a few short times in admirable yield (87–96%). The authors also explored the scope of the catalysts to catalyze the preparation of 4H-ch using diverse heterocyclic and aromatic aldehydes and reported that the reactions continued well on every type of aldehyde derivative, producing an excellent yield of products in shorter reaction time (Figure 2D) (Joshi et al., 2014).

### Ring Formation of 4H-Chromenes via Organo-Base-Catalyst

A highly appropriate and green method for efficient synthesis of 4H-ch was employed *via* the three-component based one-pot reaction of 1,3-cyclohexanedione, malononitrile, and aromatic aldehydes by using effectual organo-base-catalyst as a 2-aminopyridine. This newly synthetic protocol furnished the anticipated chromene ring in a quick times reaction with excellent yield and purity, economic advantages, ease of preparation, and recyclability of catalyst (Figure 2E) (Ramesh et al., 2016).

### Formation of 4H-Chromenes via Heterogeneous Base Catalyst Using Magnesium Oxide

By using magnesium oxide (MgO) as an extremely active heterogeneous base catalyst, pyran annulated chromene derivatives were prepared via one-pot reaction of three-component such as α-amino or α-hydroxy activated C–H acids, malononitrile, and aryl aldehydes, including 1,3-cyclohexanedione, dimedone, 1,3-dimethyl-6-amino uracil, 4-hydroxy-6-methylpyrone, 4-hydroxycoumarin, and 1,3-dimethylbarbituric acid. This catalyst was less expensive and simply obtainable, storable, and stable, and effortlessly recyclable and reusable (Figure 2F) (Xu et al., 2005).

### Ca(OH)_2_-Mediated One-Pot Synthesis for the 4H-Chromenes Bearing a Spirooxindole Skeleton

An effective and superficial one-pot synthetic strategy was followed for the synthesis of new 4H-ch bearing a spirooxindole skeleton using Ca(OH)_2_-facilitated one-pot synthesis of substituted malononitrile, isatins, and resorcinols. This reported novel procedure could provide biologically diverse molecules of 4H-ch in good to moderate yields under mild reaction conditions (Figure 2G) (Park et al., 2013).

### Ring Formation of 4H-Chromenes via Potassium Phthalimide-N-oxyl (PoPINO) as an Organo-Catalyst

A varied diversity of 2-amino-4H-chromenes with various substitutions on the 4H-ch scaffold were efficiently prepared through the multi-component approach of the one-pot reaction of an ethyl cyanoacetate, aromatic aldehyde, and miscellaneous enolizable CeH-activated acidic molecules with small loading of a new organocatalyst, called potassium phthalimide-N-oxyl (PoPINO), in aqueous condition. This method was a metal-free transition, clean, and ecologically benign method to the preparation of various 2-amino-4H-chromene derivatives (Figure 2H). This offered numerous advantages, including short reaction time, higher yields, and a straightforward and economic methodology (Dekamin et al., 2013).

### Ring Cyclization via Nitrophenylboronic Acid as a Green Catalyst and DBU Catalyst

**Figure 3:**
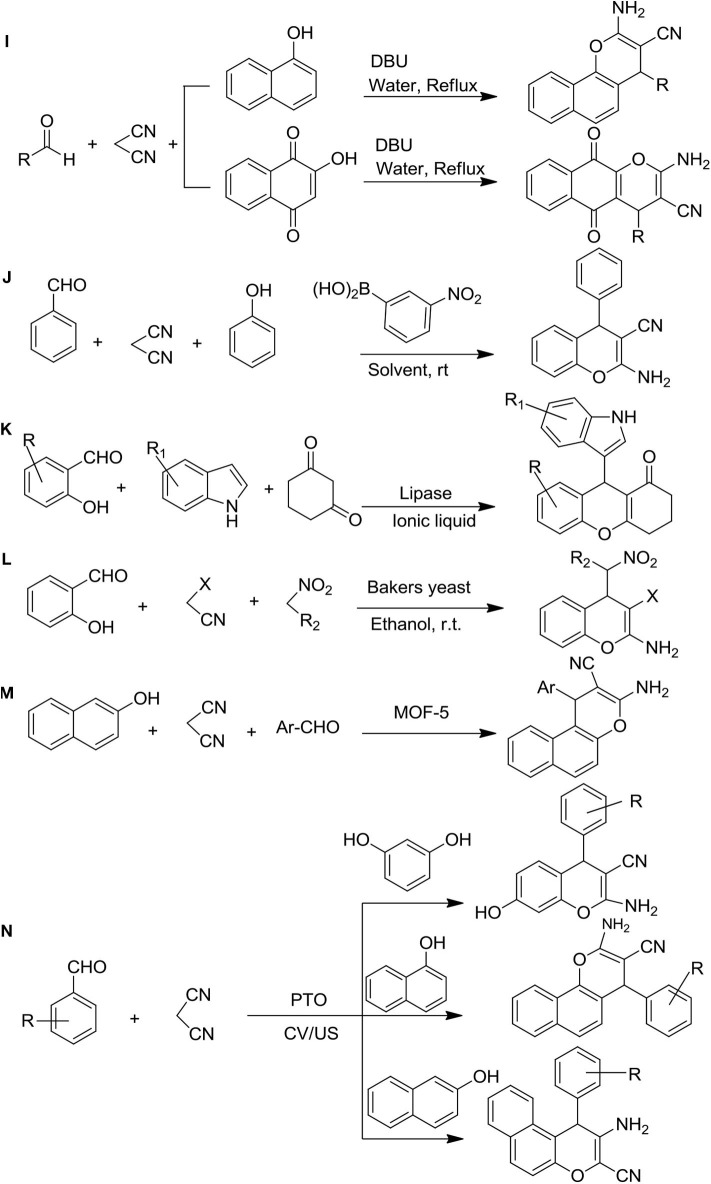
Synthesis of 4H-ch derivatives: **(I)** DBU catalyzed, **(J)** 3-nitrophenylboronic acid as a green catalyst, **(K)** lipase-catalyzed synthesis, **(L)** baker's yeast catalytic one port synthesis, **(M)** metal-organic framework catalyzed synthesis, and **(N)** potassium-titanium-oxalate-catalyzed ultrasonic synthesis.

The one-pot synthesis through DBU-catalyzed to the formation of 2-amino-4H-benzo[g]chromenes, 2-amino-4H-benzo[h]chromenes, 3,4-dihydropyrano[3,2-c] chromenes, and dihydropyrano[4,3-b]pyranes were reported from the various aldehyde, vigorous methylene containing molecules as ethyl cyanoacetate, or malononitrile and 2-hydroxynaphthalene-1,4-dione/4-hydroxycoumarin/4-hydroxy-6-methylpyrone/1-naphthol under reflux with water. The gorgeous characteristics of this procedure were the reusability of the reaction media, minor reaction environments, and shorter duration of reaction, excellent yields, and ease of product isolation (Figure 3I) (Khurana et al., 2010).

The green catalyst 3-nitrophenylboronic acid method was employed for the preparation of 2-amino-4H-chromenes through the multi-component condensation of one-pot reaction with various phenol, malononitrile, and aromatic aldehydes in ethanol. The most important feature of this reaction strategy was the mild reaction conditions, ease of the experiment, and environmental friendliness with excellent yields of the desired products, making it an attractive and efficient workup (Figure 3J) (Goswami et al., 2016).

### Lipase-Catalyzed Synthesis of Indolyl 4H-Chromenes

A new concise route of indolyl 4H-ch was developed by Zhand et al. (Figure 3K). This reaction was a three-component reaction and lipase (*Mucor miehei*) in ionic liquids was used as a catalyst. The reactant [EMIM][BF_4_] showed good reusability in this enzymatic reaction and produced higher yields of products (Zhang et al., 2017).

### Baker's Yeast Catalytic One-Port Three-Component Synthesis of 4H-Chromenes

The baker's yeast catalyzed multicomponent synthesis of substituted 2-amino-4H-chromenes was reported by Shrivas and Pratap (2019) (Figure 3L). The mixture of malononitrile, salicylaldehyde, and nitroalkanes was catalyzed by baker's yeast at room temperature with natural pH to be obtained good yields of chromenes (Shrivas and Pratap, 2019).

### Metal-Organic Framework Catalyzed Synthesis of 2-amino-4H-Chromenes

The new synthetic route for 2-amino-4H-chromenes was discovered by Arzehgar et al. (2019) using the MOF-5 catalyst (Figure 3M). For this reaction, one-pot multicomponent synthesis was followed in the presence of substituted aromatic aldehyde, malononitrile, and 2-naphthol, which led to giving an excellent yield in a short time (Arzehgar et al., 2019).

### Potassium-Titanium-Oxalate-Catalyzed Ultrasonic Synthesis of 4H-Chromenes

The ultrasonic-assisted synthetic procedure of substituted chromenes in the presence of a catalytic amount of potassium titanium oxalate dihydrate was discovered by Manake et al. (2020) (Figure 3N). This method gave a higher percentage of yield of chromenes compared to the conventional methods in less time (15 min) at 40 kHz and 40°C (Manake et al., 2020).

### Formation of 2H-Chromenes Retinoids Hybrid by Followed the Suzuki Cross-Coupling Pallado-Catalyzed Reactions

**Figure 4:**
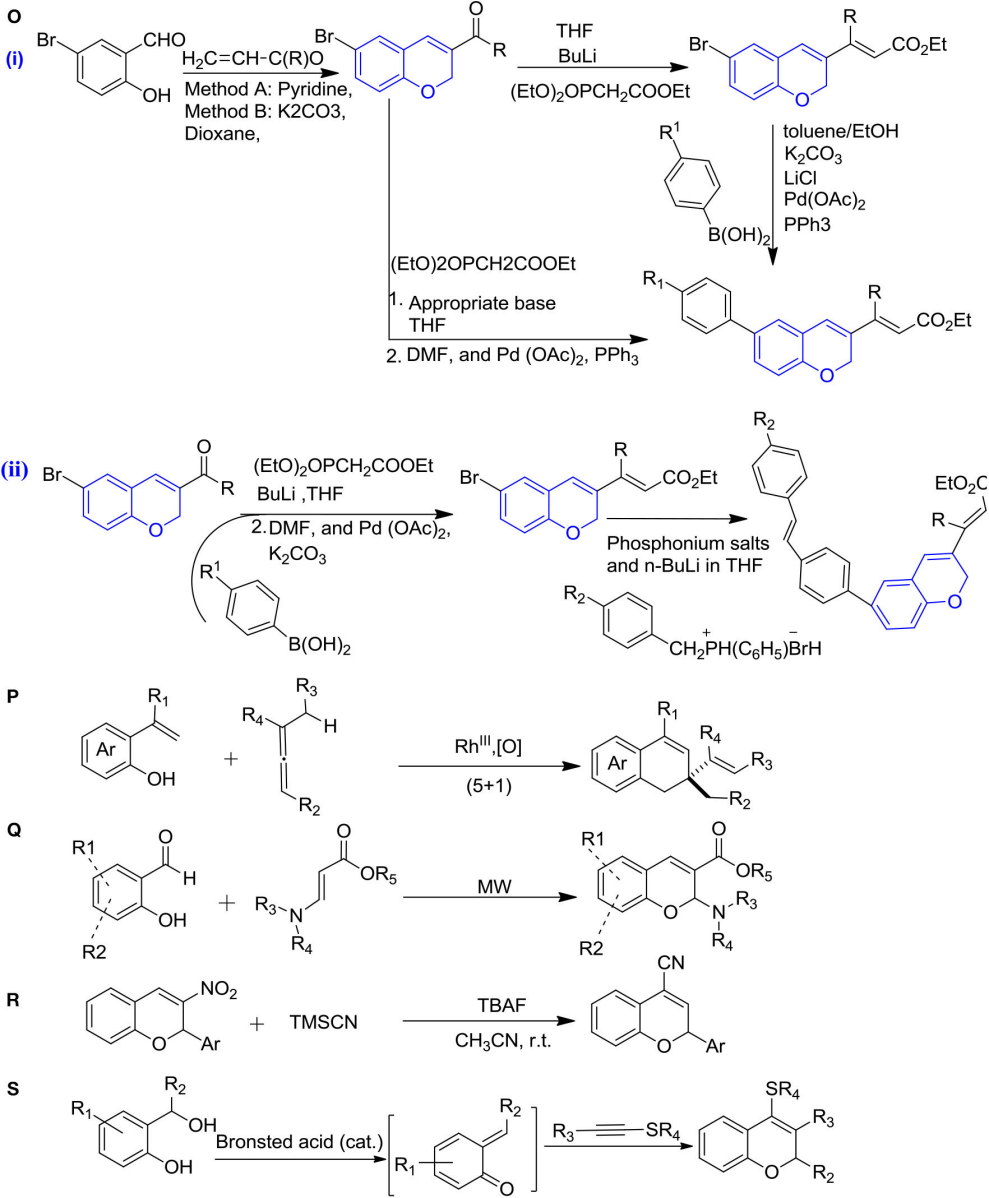
Synthesis of 2H-ch derivatives: **(O)** Witting-Horner-Emmons and Suzuki-Miyaura cross-coupling pallado-catalyzed, (i)-chromenes, (ii)- chromenes fused-retinoids hybrids; **(P)** rhodium-catalyzed (5+1) annulations; **(Q)** microwave-assisted catalyst-free synthesis; **(R)** TMSCN catalyzed Michael addition/elimination; and **(S)** unexpected [4+2] annulation of alkynyl thioethers.

An efficient synthesis of 2H-ch incorporated with retinoids was reported by Kaoukabi et al. (2019) using the witting-Horner-Emmons and Suzuki-Miyaura cross-coupling pallado-catalyzed reactions in the presence tetrahydrofuran at −78°C to 25°C and the further solvent was evaporated and replaced by DMF, Pd(OAc)_2_ and PPh3 under nitrogen. 2H-ch derivatives are generally obtained easily and in higher yields by this method (Figure 4Oi). The remarkable modification in the previous scheme was done by using BuLi, which led to giving chromene-retinoids hybrid derivatives with an efficient yield (Figure 4Oii) (Kaoukabi et al., 2019).

### Rhodium-Catalyzed [5+1] Annulations for the Formation of 2,2-Disubstituted 2H-Chromenes

For the first time, the synthesis of 2,2-disubstituted 2H-chromenes was reported by Casanova et al. (2015) from the alkenyphenols with allenes under the rhodium catalysis (Figure 4P). In this reaction, one C-H bond cleavage was involved in the alkenyl motif and further allene contributed with one-carbon cycloaddition partner, that is, (5+1) heteroannulation. The mixture of alkenyphenols and allenes were heated at 100°C in the solvent toluene with a catalytic amount of (RhCp^*^Cl_2_)_2_ and Cu(OAc)_2_.H_2_O, which led to give a higher yield of chromenes (Casanova et al., 2015).

### Microwave-Assisted Catalyst-Free Synthesis of Substituted 2H-Chromenes

The microwave-assisted catalyst-free synthesis of substituted 2H-ch from β-amino acrylates and salicylaldehydes were reported by Xia et al. (2015) (Figure 4Q). As a result, this reaction gave higher yields of chromenes in the presence of ethanol at 100°C in 1–2 h. This route of synthesis chromenes is rapid, and ecofriendly and gives a higher yield of products (Xia et al., 2015).

### Michael Addition/Elimination Pathway in the Presence of Trimethylsilyl Cyanide (TMSCN)

The 2-aryl-2H-chromene-4-carbonitriles were reported (Figure 4R) under the TMSCN catalysis in the presence of tetrabutylammonium fluoride, followed by a Michael addition/elimination pathway, which led to give the moderate yields of products (Ren et al., 2018).

### Unexpected [4+2] Annulation of Alkynyl Thioethers With Alcohols (O-hydroxybenzyl) for the Formation of 2H-Chromenes

A newly metal-free synthetic approach for the synthesis of polysubstituted 2H-ch was reported by Bu et al. (2020) via unexpected [4+2] annulation of alkynyl thioethers with alcohols (o-hydroxy benzyl), which led to a high yield of products under the catalysis by Triflimide (HNTf_2_) as a highly versatile super Brønsted acid in the presence of 1,2-dichloroethane at room temperature (Figure 4S) (Bu et al., 2020).

## Biological Activities of 2H/4H-Chromenes

Various compounds derived from the 2H/4H-ch heterocyclic ring represent a key element of medicinal chemistry owing to their productive medicinal values. For the most part, unexplored heterocyclic molecules derived from the 2H/4H-ch ring acquire a diversity of pharmacological profiles, ranging from antitumor and, anticonvulsant activities on the one hand, although, on the pathogenic side, they are similarly significant owing to their antibacterial activity. Therefore, 2H/4H-ch molecules have an immense and broad range of beneficial activities.

### Anticancer Activity

**Figure 5:**
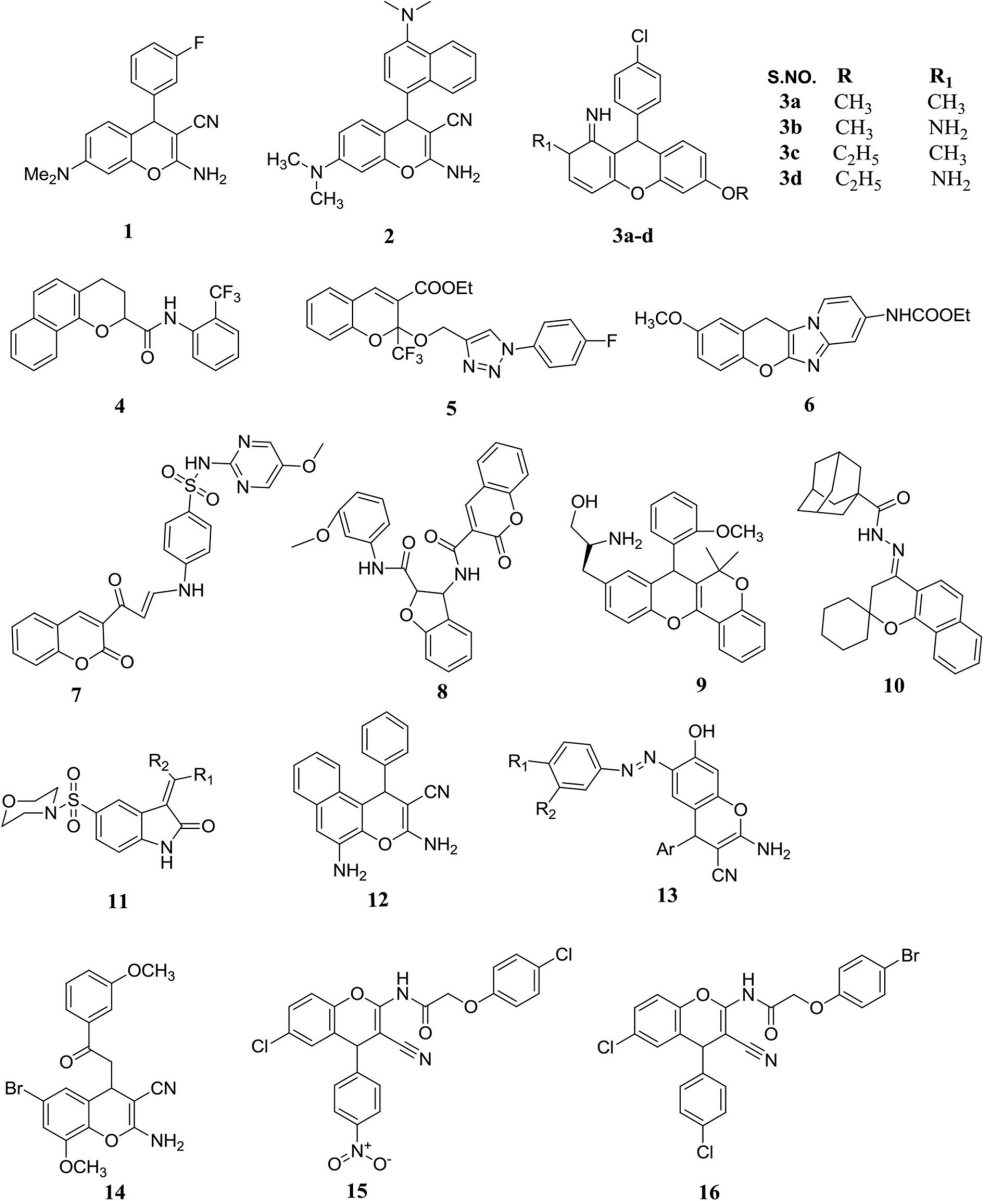
Substituted potent chromenes possess significant anticancer potency.

A small library of 2-amino-4-aryl-3-cyano-7-(dimethylamino)-4H-chromene derivatives were prepared using an aromatic aldehyde, 3-(dimethylamino) phenol, and malononitrile with piperidine in ethanolic solution (Figure 5). They determined the cytotoxic activities of the prepared molecules against six human tumor cells followed by a well-established assay namely 3-(4,5-dimethylthiazol-2-yl)-2,5-diphenyl tetrazolium bromide (MTT). Compound **1** was found to be the most active compound with a 50% growth inhibition (IC_50_) value of less than 1 μM. Through this study, the authors reported that substitution on the second, third, and fourth positions of the 4-aryl-4H-chromenes produced potent antitumor activity. In addition, that the halogenations was substituted on the third position responsible in a great intensification in antitumor potency, but substituted on the 3-position by F or Br generally afforded better cytotoxicity (Vosooghi et al., 2010).

Inspired by the potency of molecule GRI-394837 and its derivatives, an attentive set of newly chromene derivatives were synthesized in a single step *via* the microwave radiation technique and tested for their anticancer activity. As a result, chromene **2** was found to be the most active toward the A172 glioma cells with IC_50_ 7.4 nM and also showed a weak tubulin polymerization inhibition but with very strong cytotoxicity in cellular assays. These outcomes powerfully suggested that the newly chromenes should be studied extra to explore the molecular insights into the anticancer activity (Patil et al., 2012). Inspired by microtubule-interfering properties of 4-aryl-4Hchromene derivatives, Kandeel et al. (2013) combined this nucleus with five-, six-, and seven-membered heterocyclic moieties to examine the effects of a novel series of compounds. They selected 10 newly synthesized molecules for an antitumor evaluation against the MCF-7 breast cells, and most of them exhibited excellent activity compared to colchicine as a positive control. The compound **3** was found to be a more potent antitumor agent toward targeted cancer cell lines (Kandeel et al., 2013).

A novel class of substituted chromenes was constructed with the help of the 6-hydroxy-7-methoxy-chroman-2-carboxylic acid phenyl amide moiety (KL-1156) and tested their activity against a nuclear factor kappa B (NF-kB) and targeted cancer cell lines. The chromene moiety of KL-1156 was modified into four parts derived with 3- and 4-dihydro substituted 2H-benzo[h]chromene for their SAR structure features. From the SAR studies, that numerous novel N-aryl, 3, 4-dihydro substituted 2H-benzo[h]chromene-2-carboxamides were reported with excellent inhibitory activity against NF-kB and displayed better antiproliferative profile than the parental molecule KL-1156. In addition, compound **4** exhibited the best inhibitory activity on LPS-induced NF-kB based transcriptional property and also showed excellent antitumor potency against NCI-H23 lung cells compared to KL-1156 (Choi et al., 2014).

A new series of isoxazole and 2-(1,2,3-triazolylmethoxy), functionalized 2H-ch were synthesized by a cyclization reaction between ethyl-4,4,4-trifluoroacetoacetate and salicylaldehyde resulted in the formation of second hydroxy-substituted ethyl contains 2-(trifluoromethyl)-2H-chromene-3-carboxylate. All the compounds were tested for the cytotoxic evaluation toward four different targeted human cancer cells, where compound **5** exhibited promising anticancer activity with IC_50_ < 20 μM (Reddy et al., 2014).

New chromenes containing fused imidazo[1,2-a] pyridine motifs were reported and evaluated them for their antiproliferative effects in human colon HCT116 cancer cell lines. They reported that the carbamate group in the eighth position of pyridine ring containing molecule **6** was the most-effective molecule of the synthesized series and induced significant cell cycle detention at both the G2 and S phases and also showed the cell death by the apoptosis. They also showed that this activity was due to the remarkable induction of caspase-mediated apoptosis in a p53-independent manner (Lima et al., 2015).

Twenty novel chromene derivatives carrying different sulfonamide moieties were reported and tested for their *in-vitro* antitumor activity toward breast cancer cells (T47D). Maximum synthesized molecules exhibited good to reasonable cytotoxicity (IC_50_ 8.8–108.9 μM). In particular, compound **7** (IC_50_ 8.8 μM) exhibited higher cytotoxicity compared to doxorubicin (IC_50_ 9.8 μM). To determine the molecular mechanism of this activity, they tested the effects of the most potent compounds on the aromatase activity. They reported that most of them had a significant inhibitory effect on the aromatase activity. Moreover, the authors also investigated the molecular docking study, and they found a probable interaction of the potent derivatives with the aromatase enzyme (Ghorab et al., 2016).

The virtual screening was carried out of a chromene-based molecular database of the small compounds using physic-chemical, molecular docking and absorption, distribution, metabolism, excretion, and toxicity (ADMET) profiling. Potential hit compounds were identified among them. To validate the lead compounds, they carried out molecular dynamics simulations and related analysis and reported that the lead compound **8** (PubChem CIDs: 16814409) a potential candidate for tubulin inhibition (Aryapour et al., 2017).

A miscellaneous chromene containing xanthene hybrids molecule library were prepared via an intramolecular Friedel crafts synthesis of the arenoxy carbinols. Here, the authors incorporated the tyrosine amino acid onto the xanthenes scaffold with hydrophilic functionalities. A precise structure-based screening showed that newly modified tyrosine containing chromene-xanthene hybrids molecule exhibited excellent potential toward MDA-MB-231 and MCF-7 cells. All collective outcomes suggested that the lead molecule **9** manifested noteworthy arrested cell cycle at the G1 phase and induced the apoptosis in MDA-MB-231 cell lines (Kumar et al., 2018).

Chromene-containing molecules were reported with either established or hopeful pharmacological potency and showed that the chromone motif is a scaffold for the advance in the discovery of new medicines. These molecules confirmed better drug-like characteristics and a wide binding affinity for various significant enzymes. Simultaneously, the authors investigated the possibility of its therapeutic effect focusing generally on the anti-inflammatory potency that may move to additional central disease targets such as diabetes, brain disorders, and cancer (Silva et al., 2018).

Spirobenzo[h]chromenes were prepared and tested for their antitumor potency against HT-29 (human colorectal cancer cells), MCF-7 (human breast cancer cells), and A549 (human lung cancer cells) cells *in vitro* by using an MTT assay. Among the molecules synthesized, eight exhibited a better anticancer profile than sorafenib, with IC_50_ 1.78 and 5.47 μM. Representative compounds were selected for further molecular study through EGFR, B-RAF, and tubulin polymerization method. Molecule **10** was the greatest effective EGFR inhibitor, IC_50_ 1.2 μM, and exhibited a better inhibitory effect against tubulin polymerization. They also performed docking of these molecules and investigated their imaginable interactions into the active binding pocket of B-RAF kinases both and EGFR (Figure 5) (Abdelatef et al., 2018).

Fourth-position hydroxy-substituted 5′-(morpholinosulfonyl) spiro[chromene-2,3′-indolin]-2′-one and 3-phencyclidine-2-oxindoles were prepared by a reaction of 5-morphilinosulfonyl isatin with acetophenones followed by treatment with acetic acid. All the compounds were tested for their antitumor potency toward three tumor cells, such as MCF-7, HCT-116, and HepG-2, by using an SRB (sulforhodamine B) assay. Compound **11** exhibited a wide range of antitumor effectiveness on these tested cancer cell lines with IC_50_ ranges less than 10 μM and arrested the cell cycle at the G_0_-G_1_ phase. This compound also exhibited extra effect toward EGFR than Lapatinib with IC_50_ ranges from 0.019 μM, while the IC_50_ value of Lapatinib was 0.028 μM (El-Sharief et al., 2019).

A new series of 4H-ch-based azo chromophores derivatives were reported and tested for their anticancer activities. As a result, these derivatives exhibited anticancer potency against HCT-116, MCF-7, and HepG-2 cancer cell lines. Importantly, compounds **12** and **13** showed higher potency in the antitumor assay with an IC_50_ range of 0.3 to 2 μg/mL (Afifi et al., 2017b).

Also, 4H-ch analogs were synthesized and tested their anticancer activities against the targeted cancer cells. As a result, compound **14** showed remarkable anticancer activity against MCF-7 and Hs578T. Further evaluation showed that compound **14** has satisfactory migratory cell reduction capacity and reduced cell proliferation by arresting cells in the G2/M phase. Additionally, compound **14** exhibited an inhibitory effect against tubulin dynamics. Therefore, it may be a promising compound for inhibiting tubulin polymerization in the cancer cells (Pontes et al., 2018).

Substituted 4H-ch were synthesized and evaluated their potency toward the HT29 human colon cancer cells. Compounds **15** and **16** exhibited the maximum inhibitory effect against HT-29 cells than the other derivatives of their series. According to *in-silico* studies, **15** and **16** showed a stronger interaction with many H and π-bonds. On the other hand, both studies strongly correlated and supported the author's hypothesis. Therefore, these compounds might have better potency to bind with the cancer protein targets (Chauhan et al., 2020).

A novel series of substituted-6-methoxy-4H-benzo [h]chromenes was reported by Ahmed et al. (2019) and evaluated their anticancer potency. The surprising result was shown with the MCF-7 cell line, compounds **17**, **18**, **19**, **20**, **21**, and **22** showed the significant inhibition of MCF-7 (IC_50_ 1.2–5.5 μg/mL). On the other hand, compounds **22** and **17** exhibited the potent inhibition against HCT-116 cell line (IC_50_ 1.3 and 1.9 μg/mL, respectively) compared to vinblastine (IC_50_ 2.6 μg/mL). Also, compounds **18**, **19**, **17**, **22**, **23**, **24**, **25**, and **26** were observed to be the considerable active against the HepG-2 cell line. Moreover, compounds **19**, **21**, **17**, **20**, **18**, and **22** were subjected to further evaluation for their c-Src kinase inhibitory activities. Among them, compound **21** exhibited highly significant activity (IC_50_ 0.1432 μM) against c-Src kinase. Overall, the studies suggested that the substitution of lipophilic electron-withdrawing such as -Br or an electron-donating likely -OCH_3_ in the naphthalene motif is highly suitable than the unsubstituted naphthalene to the inhibition of c-Src kinase (Ahmed et al., 2019).

An efficient catalyst-free, one-pot synthesis was employed for the preparation of new 4H-ch and evaluated their anticancer potency against the targeted cancer cells. As a result, compounds **27**, **28**, and **29** showed more than 50% activity against A549 tumor cell line, while no significant cytotoxicity was found against the MCF-7 and HT29 cell lines. Additionally, all chromenes showed the potent inhibition of α-glucosidase compared to acarbose (IC_50_ 4.90 mM). In the tyrosinase inhibitory assay, all compounds showed moderate inhibition of tyrosinase in the range of IC_50_ 3.50–12.20 mM. Besides, *in-silico* molecular docking studies revealed the atomic interaction of chromenes with the binding site of α-glucosidase and tyrosinase. The molecular docking analysis suggested that hydrogen bonding, π-π stacking, hydrophobic, metal, and π-cation interactions were important between synthesized chromenes and targeted enzymes to produce significant binding with enzymes (Dinparast et al., 2020).

New 2-substituted 4H-benzo[h]chromenes and 7H-benzo[*h*]chromeno[2,3–d] pyrimidines were prepared by Alblewi et al. (2019b) and assessed their *in-vitro* anticancer activity. As a result, compounds **30** and **31** (IC_50_ value 0.45 and 3.8 μg/mL, respectively) showed 13.6 and 1.6 times more cytotoxicity than vinblastine (IC_50_ 6.1 μg/mL), though compound **30** has the similar activity as doxorubicin, with an IC_50_ value of 0.4 μg/mL against MCF-7. Moreover, overall structural observation of 4*H*-benzo[*h*]chromenes showed that presence of hydrophobic group likely -N=CHOEt, -NHCOMe, and -N=CHNH_2_ at the second position with -CN-3 or presence of pyrimidine motif with the hydrophobic group as Me-9 or NH-8 at the second and third positions had a significant impact for the enhancement of inhibitory activity toward the cancer cell line than other substitution of this series. Additionally, compounds **32**, **33**, and **30** showed the cancer cell arrest in the S and G2/M-phases and also exhibited the inhibition of tumor cell migration and invasion. All these compounds showed early and late apoptosis after the exposure under Annexin V/PI double staining. Thus, an *in-vitro* study showed that compounds **32**, **33**, and **30** had the potency to inhibit cancer, but in the respect of *in-vivo* study, further investigations are required to clarify the actual potency inside the systemic circulation (Alblewi et al., 2019b).

Also, a series of newly 2-glyco-3-nitro-2H-chromenes were synthesized Luque-Agudo et al. (2019) using oxo-Michael-Henry-dehydration reactions and tested for their activity against the cancer cell lines. Regarding cancer cell line inhibition, compounds **34**, **35**, **36**, and **39** [growth inhibitory 50% (GI_50_) 1.9, 1.7, 2.6, and 3.1 μM, respectively] displayed good GI_50_ against the WiDr multidrug resistance cells. Improvement in the anticancer potency was found with substitution of halogen or methoxy group at the C-6 position of chromene scaffold. These findings may be helpful to the future study involving the use of selective target inhibition in cancer. Although the mixture of **35+38** and **36+37** has been identified as a potent inhibitor of the targeted cell line, these compounds should be tested for their anticancer potency over *in-vivo* cancer (Luque-Agudo et al., 2019).

Azo-sulfa fused chromone motifs were synthesized and tested for anticancer activity and also inhibition of a class I histone deacetylase (Okasha et al., 2019). As a result, all compounds in this series showed remarkable cytotoxicity against the targeted cancer cell lines. A significant and encouraging anticancer activity was found to the compound **40**, **41**, with an IC_50_ value of 1.8 and 1.7 μM toward the MCF-7 and HepG-2 cell lines. The compound **42** showed the inhibition *in-vitro* of both histone deacetylase-1 and 2, while compound **43** exhibited effective inhibition against histone deacetylase-1 over histone deacetylase 2 and 8. This series of molecules is still on the primary stage of development; therefore, various structural modifications are possible in azo-sulfa-fused chromone motifs to enhance the potency of these molecules (Okasha et al., 2019).

By inspired the previously reported anticancer compounds of a series of 4-aryl-4H-chromene (Knox et al., 2007), Carr et al. (2020) synthesized a series of new compounds by the addition of basic aryl ethers at the C-4 position and C-3 modified ester substitution in the benzopyran ring and tested their desired effectiveness against estrogen receptor (ER) as antagonists in MCF-7 tumor cell line (Carr et al., 2020). Among them, compound **46** (IC_50_ 2.65 μM) was found to be the potent anticancer agent compared to tamoxifen (IC_50_ 4.12 μM). Regarding ER inhibition, it was observed that either substitution of pyrrolidine, piperidine, or substitution of morpholine on the chromenes motif led to improve binding potency with ERα. The ethyl **44**, methyl **45**, and *tert*-butyl **46** esters of piperidine-containing compounds were found to be more selective toward ERα. The morpholine side-chain-contained compounds, the ethyl ester **48**, and the methyl ester **47** showed a high specificity with ERα and ERβ, respectively. This observation proved the authors' assumption that the substitution of basic aryl ethers or modified substitution with ether could help to enhance the binding selectivity toward the ER. These molecules have a great interest in the further study including the use of a selective ER inhibition *in-vivo* study (Carr et al., 2020).

Two novel series of H-ch and 4H-benzo[h]chromenes compounds were synthesized and tested for their anticancer potency against the targeted HCT-116, MCF-7, and HepG-2 cancer cell lines (Afifi et al., 2017a). As a result, compounds **49**, **50**, **51**, **52**, **53**, **54**, and **55** were found to be active with an IC_50_ value range from 0.3 to 3.78 μg/mL. The azo-substituted chromenes disappointed the assumption related to improving anticancer activity toward HCT-116, MCF-7, and HepG-2 cell lines except compound **55** of the same series. Additionally, compounds **50** and **51** improved the caspase activity in the targeted cell line. In acute toxicity studies, compound **51** was ineffective to release the *Lactate dehydrogenase* at 100 μM concentration. These molecules are still on the primary stage of development; therefore, possible structural modification is needed for the improvement of the potency of these molecules (Afifi et al., 2017a).

Cationic organoiron complexes incorporating with chromenes were designed and further synthesized *via* Steglich esterification with nucleophilic substitution reaction for the desired yield of targeted molecules. Additionally, these molecules were tested to evaluate their effectiveness for anticancer activities. As a result, compounds **56** and **57** (IC_50_ 15.6 and 29.3 μM) showed the remarkable cytotoxicity in the MCF-7 cell line. Also, SAR studies in this series might be helpful to identify the lead molecule from this series as an anticancer (Abd-El-Aziz et al., 2020).

**Figure 6:**
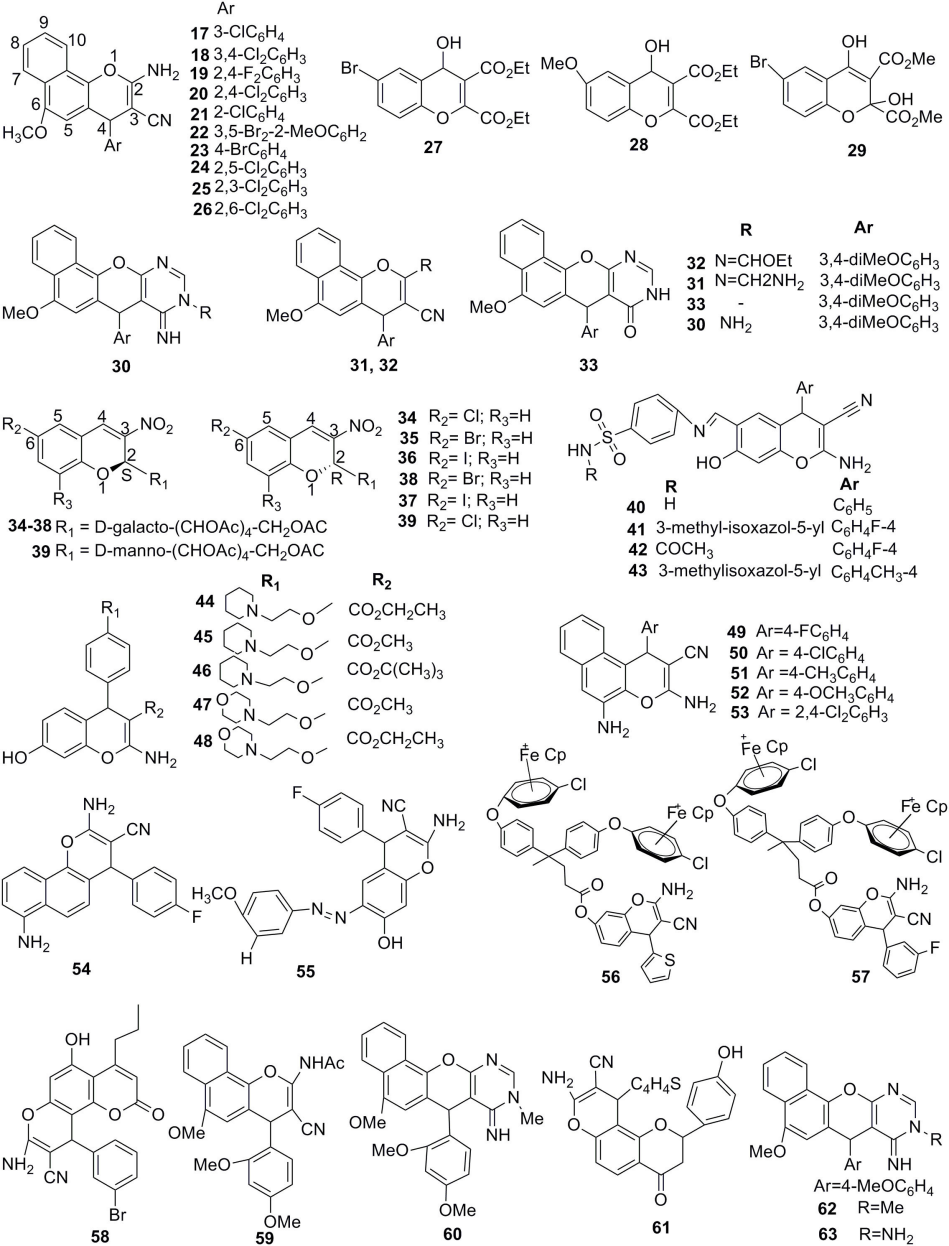
Potent 2H/4H-ch analogs for significant anticancer activities.

Fluorescent 5,7-dihydroxy-4-propyl-2H-chromen-2-ones were synthesized and tested for their anticancer potency. This series of compounds exhibited the considerable IC_50_ value in the range 2.9 to 11.8 μg/mL against HCT-116, HEPG-2, and A-549 cell lines, but against MCF-7 cell line, compound **58** showed the remarkable inhibition with IC_50_ of 0.86 μg/mL compared to standard drug doxorubicin (Abd-El-Aziz et al., 2016) (Figure 6). Due to a better potency of compound **58** toward the inhibition MCF-7 cell line, it might be considered for future study to address the molecular mechanism of this molecule for inhibiting cancer.

A series of novel heterocyclic incorporated 4H-benzo[h] chromenes were designed and synthesized and evaluated for their anticancer specific potency against targeted cancer cell lines (Alblewi et al., 2019a). As a result, compounds **59** and **60** showed higher anticancer activity toward the all targeted cancer cell lines with IC_50_ ranges of 0.7 to 3.0 μg/mL and 0.8 to 1.4 μg/mL, respectively. Further cell cycle phase distribution studies revealed that compound **59** exhibited the cancer cell arrest at the S and G1 phases in the MCF-7 and HepG-2 cells, while both the S and G2/M phases were arrested in HCT-116 cell line. Compound **60** showed the cancer cell arrest at the G1 phase. Moreover, compounds **59** and **60** did not exhibit any necrosis; therefore, cell death found primarily by the induction of apoptosis. Apoptosis was induced because compounds **59** and **60** amplified the significant level of caspase3/7. These compounds also significantly influenced on DNA fragmentation. Therefore, compounds **59** and **60** trigger cell apoptosis by the activation of caspse3/7 and executioner DNA fragmentation in cancer cells. Additionally, these compounds also exhibited a substantial reduction in cell invasion and cell migration parentage (Alblewi et al., 2019a). These compounds are potent lead molecules for the development of anticancer drug molecules.

Novel flavanones incorporated with chromenes were synthesized by the one-step multicomponent reactions, and their anticancer activities were estimated against the HCT-116, HepG-2, A-549, and MCF-7 cell lines. Among them, compound **61** exhibited remarkable potency toward all targeted cell lines in the range of IC_50_ 1.08–2.42 μg/mL. It is needed to discover the action mechanism of molecule **61** and to develop an effective lead molecule for the treatment of cancer (Assirey et al., 2020).

The new series of benzo[h]chromenes was synthesized and tested their anticancer activities. As a result, compounds **62** and **63** were found to be most actives against the targeted cell lines with the IC_50_ values in the range 0.9 μg/mL and 0.8–1.1 μg/mL, respectively. Interestingly, incorporation of hydrophobic groups at the motif of chromene fused by pyrimidine ring with 2,3-positions and lipophilic introduction is more appropriate to this series of compounds to enhance the anticancer potency (Okasha et al., 2017).

### Anticonvulsant Activity

Two 2H-ch-containing hydrazones were synthesized and tested for their anticonvulsant potency and neurotoxicity followed by maximal electroshock-induced seizure (MES) tests and subcutaneous pentylenetetrazol (scPTZ) in ICR mice. These molecules exhibited dose-dependent protection and a tendency to alleviate the mortality in the MES test, but they were less effective than phenytoin. Compound **64** offered strong protection against the MES test, suggesting its ability to inhibit the seizure spread, while compound **65** showed higher anticonvulsant activity in the scPTZ test. Both compounds manifested minor neurotoxicity in their highest administered dose of 300 mg/kg (Angelova et al., 2016).

A new series of the 3-(5-pheny-1,3,4-oxadiazol-2-yl)-2H-chromen-2-ones were synthesized by (Sudha et al., 2018) and evaluated their potency against convulsant using MES and scPTZ. Among them, compound **66** exhibited highly significant activity in both assigned models (Sudha et al., 2018).

### Antimicrobial Activity

2-cyano-4-oxo-3-phenyl-3,4-dihydro-2H-furo[3,2-c]chromene-2-carboxylates were synthesized using a one-pot multi-component reaction strategy. The *in-vitro* antimicrobial potency of these compounds was carried out against different Gram-negative and positive bacterial strains. As a result, compound **67** had promising activity against *Klebsiella planticola* MTCC 530 and *Micrococcus luteus* MTCC 2470 bacterial strains. On the other hand, compound **68** exhibited excellent activity against *Bacillus subtilis* MTCC 121, *Escherichia coli* MTCC 739, *Micrococcus luteus* MTCC 2470, and *Klebsiella planticola* MTCC 530, and showed reasonable potency toward *Candida albicans* MTCC 3017 and *Staphylococcus aureus* MTCC 96 compared to Ciprofloxacin (Kale et al., 2016).

In another study, a series of indolyl-4H-chromene-phenyl prop-2-en-1-one derivatives were prepared by Subbareddy et al. (2018) *via* regioselective nucleophilic substitution. They evaluated theirs *in-vitro* effective against Gram-negative bacteria (*E. coli* and *Klebsiella*) and Gram-positive (*B. subtilis* and *S. aureus*) bacterial by using an agar method. Among them, molecules **69** and **70** exhibited significant antibacterial activity (both MIC 9.3 μg/mL) against *S. aureus* compared to the standard drug (Subbareddy et al., 2018).

A well-organized one pot three-component reaction for the synthesis of 2-amino-4H-chromenes were employed by Aminkhani et al. (2019) by using a range of aromatic aldehydes, orcinol, and malononitrile at 25°C in CH_2_Cl_2_ with a catalytic amount of triethylamine. They performed antimicrobial screening against Gram-positive bacteria including *Bacillus anthracis* and *S. aureus*, using a disk method, minimum inhibitory, and bactericidal concentrations approach. Among them, some of the compounds synthesized showed potent antibacterial activities (i.e., **71a, 71b, 71c, 71d**, and **71e**) toward Gram-positive bacteria, such as *B. anthracis, Enterococcus faecalis*, and *S. aureus*, and Gram-negative bacteria, such as *Pseudomonas aeruginosa, Salmonella paratyphi B*, and *E. coli*. All compounds tested slowed down the growth of *S. aureus* and *B. anthracis* but did not affect *E. faecalis* and Gram-negative bacteria (Aminkhani et al., 2019).

1H-1,2,3-triazole-tethered-4H-chromene-containing D-glucose conjugates were synthesized by using propargyl ethers and tetra-O-acetyl-b-D-glucopyranosylazide. These molecules were evaluated for *in-vitro* activity against microorganisms such as *B. subtilis, S. aureus*, and *S. epidermidis*. Among them, compounds **72a, 72b, 72c, 72d**, and **72e** showed MIC values in the inhibition range of 1.56 to 6.25 μM. Compounds **72e** (MIC 1.56 μM), (**72c**) (MIC 3.12 μM), and **72d** (MIC 6.25 μM) were better effectiveness against *B. subtilis*, and compounds **72a, 72d, 72e** exhibited considerable antimicrobial potency toward *S. aureus* with MIC ranges of 6.25, 3.12, and 3.12 μM, respectively. Molecules **72f, 72g, 72h**, and **72i** showed the highest potency toward *S. epidermidis* (MIC ranges of 3.12, 6.25, 1.56, and 3.12 μM, separately). On the other hand, the aforementioned triazoles showed similar inhibitory activity to ciprofloxacin but less than vancomycin, except for **72f, 72d, 72e** (MICs 1.56 μM) (Thanh et al., 2019).

By using multicomponent reactions, two novel series of H-ch and 4H-benzo[h]chromenes compounds were synthesized and tested for their antibacterial and antifungal activities (Afifi et al., 2017a). As a result, compounds **73a** and **73b** showed the remarkable inhibition toward the targeted Gram-positive and fungi with MIC range 0.007 to 0.49 μg/mL and zone inhibition range 18 to 30 mm. On the other side, compounds **74a** and **74b** exhibited higher activity against the Gram-positive and fungi with a range of zone inhibition 10–23 mm. Compounds **75a**, **75b**, **75c**, and **75d** showed the higher MIC value against the Gram-positive in the range 0.06 to 0.49 μg/mL and antifungal activity with range 0.49 to 1.95 μg/mL (Afifi et al., 2017a). Furthermore, SAR studies of these series should be continued to identify the lead molecule for future drug development.

The synthesized cationic organoiron complexes incorporated with chromenes were tested for their antimicrobial activities (methicillin-resistant *S. aureus*, vancomycin-resistant *Enterococcus faecium* and *S. warnerii*). As a result, compounds **76** (IC_50_ 2.40 and 2.04 μM) exhibited considerable inhibition against *S. aureus* and *S. warnerii*, while compound **57** (IC_50_ 2.12 μM) showed the remarkable inhibition against *E. faecium* (Abd-El-Aziz et al., 2020).

Fluorescent 5,7-dihydroxy-4-propyl-2H-chromen-2-ones were tested for the antimicrobial activities by serial dilution method (Abd-El-Aziz et al., 2016). As a result, compounds **77a** and **77b** of this series showed the considerable zone inhibition against both Gram-positive and negative bacteria and also showed effective inhibition against the fungi. The MIC was further investigated where compounds **77b** and **77c** were found to be active against both Gram-positive and negative bacteria and also effective against fungi with MIC values in the range 0.49 to 3.9 and 1.95 to 31.25 μg/mL, respectively. A molecular docking study revealed the binding interaction of targeted molecules with topoisomerase II DNA gyrase B binding pocket. Interestingly, SAR of this series of compounds showed that substitution of the electron-withdrawing group on the targeted molecule contributes improvement of antimicrobial activities, particularly against Gram-negative bacteria (Abd-El-Aziz et al., 2016).

Benzochromenes bearing aryl azo scaffolds were synthesized and tested their specific activity against the microorganisms. As a result, compound **78** showed considerable activity against the *P. aeruginosa* and *E. coli*, while compound **79** showed the remarkable antibacterial activity against the Gram-positive bacteria. Moreover, compound **80** showed the remarkable inhibition against the fungi compared to the reference drug. Future study is needed to address the SAR of this series of molecules to develop effective lead molecules for the discovery and development of chromenes containing drugs for antibacterial activities (Afifi et al., 2019).

### Antiacetylcholinesterase Activity

A novel series of chromene derivatives linked to the 1,2,3-triazole motif were synthesized and tested for their antiacetylcholinesterase potency. As a result, compound **81** exhibited better antiacetylcholinesterase activity with IC_50_ of 15.42 μM. Moreover, this compound also showed the neuroprotective effect against hydrogen-peroxide-induced cell death model in the PC12 cells. Through kinetic and molecular docking studies, authors established the dual π-π binding between a drug molecule and both the peripheral anionic site and the catalytic active site of acetylcholinesterase (Saeedi et al., 2017). These findings supported that compound **81** is an effective molecule to inhibit the acetylcholinesterase.

### Antihyperglycemic Activity

**Figure 7:**
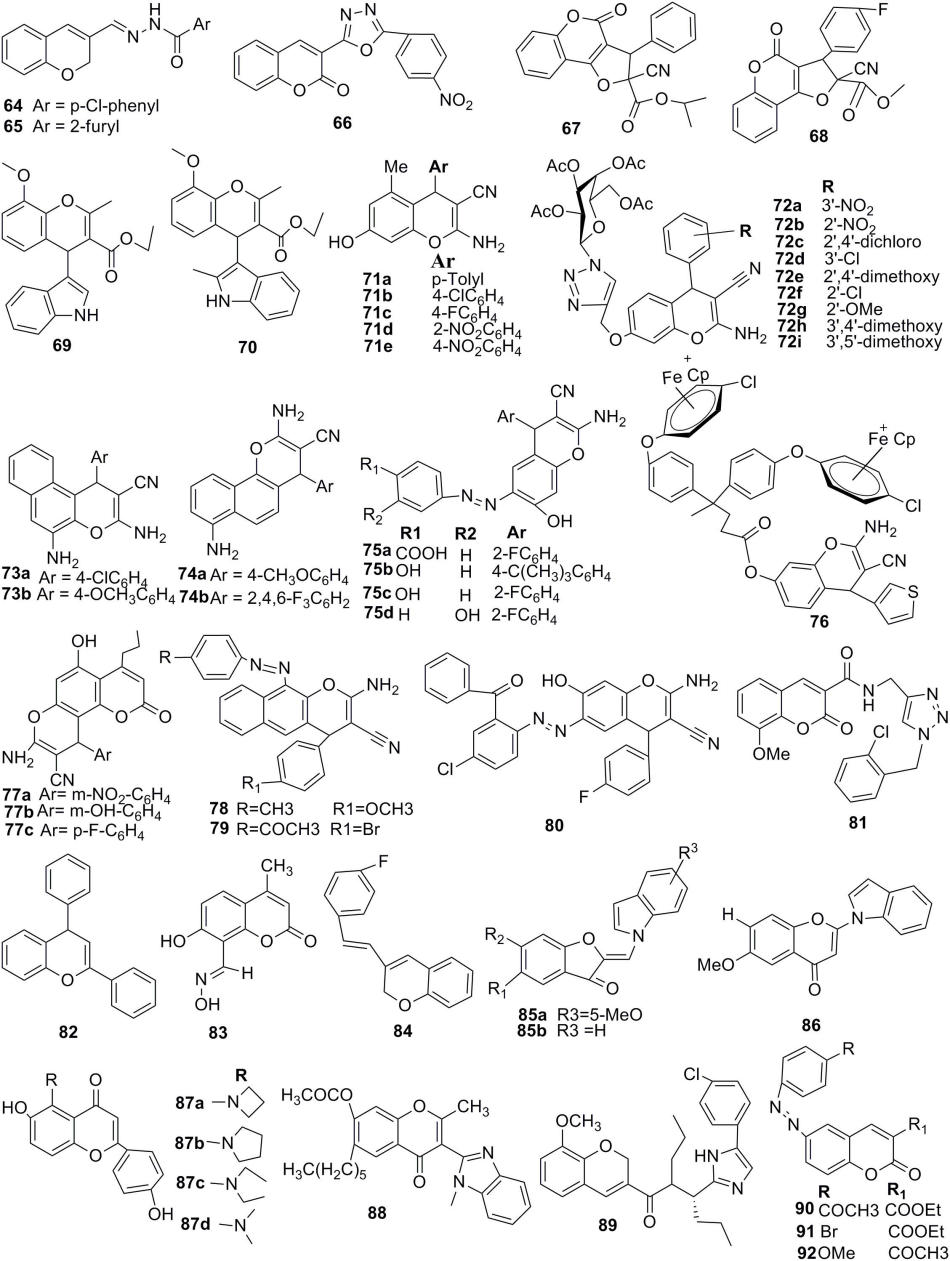
Potent 2H/4H-ch derivatives possess various significant biological activities.

Arylchromenes as naturally occurring flavonoids analogs were reported with the outstanding inhibitory potential of α-glucosidase (Figure 7). As a result, that the 2-aryl-4H-chromene core **82** retained its pharmacophoric properties while being readily available synthetically. They also concluded that a lead compound identified by screening of all the derivatives inhibited the α-glucosidase enzyme yeast (IC_50_ 62.26 μM) and prevented the postprandial hyperglycemia (Spasov et al., 2019).

A series of the seventh position substituted hydroxy-4-methyl-2-oxo-2H-chromene-8-carbaldehyde oxime (7-oxime) molecules were reported and evaluated for their *in-silico* inhibitory potency via docking studies. As a result, compound **83** showed a better binding affinity with both human α-amylase and α-glucosidase. However, the aforementioned compound could be utilized as an additional molecule for the diabetes pre-management (Ghazvini et al., 2018).

### Monoamine Oxidase Inhibitors

A newly series of 3-(E)-styryl-2H-chromenes were synthesized and screened them against the MAO A and B. None of the tested compounds showed inhibitory activity against MAO-A at 10 μM. In contrast, most of the compounds in the series had strong inhibitory potential particularly against MAO-B at the same concentration. Compound **84** bearing fluorine at the position R_3_, exhibited most promising potency (IC_50_ 10 nM) and was 22-fold extra effective than the positive control, pargyline. They claimed that this was the primary statement recognizing 3-(E)-styryl-2H-ch as selective and effective MAO-B inhibitors and suggested that the 3-(E)-styryl-2H-chromene scaffold could be an advantageous molecule for the developing of newly MAO inhibitors (Takao et al., 2018).

In another study, Takao et al. (2019) designed and synthesized the substituted-4H-chromen-4-one derivatives and tested their specific activity toward the inhibition of MAOs (Takao et al., 2019). As a result, compounds **85a** and **85b** exhibited a higher inhibition against the MAO-A compared to pargyline as a standard drug. Interestingly, compound **86** showed more specific higher inhibition of MAO-B, with an IC_50_ value of 0.0026 μM. From these outcomes, it was concluded that methoxy substitution at R1 position on the ring A of flavonoids was responsible to increase the MAO-A selective inhibition, while methoxy group at R2 position was responsible to increase the MAO-B selective inhibition. This series of compounds are needed for more substitution to prove the potent lead for design effective MAOs inhibitors (Takao et al., 2019).

### Antituberculosis Activity

Inspired by the crystal structure of polyketide synthase (pks13) inhibitor TAM16, the morphing scaffold was incorporated to design and synthesize a series of 4H-chromen-4-one derivatives and tested their specific potency toward the inhibition of tuberculosis (Zhao et al., 2020). As a result, compound **87a** exhibited higher activity with MIC of 0.55 μg/mL, while compound **87b** also so remarkable effect against the *M. tuberculosis* H37Rv (drug-resistant of stains). In contrast, ring-opened diethylamine and dimethylamine compounds **87c** and **87d** disappointed because they did not show any activity. The cell toxicities study of compounds **87b** and **87a** were tested and selectivity index values were also calculated using the ratio of IC_50_/MIC, where compound **87d** showed the low toxicity and high selectivity index values. Interestingly, compound **87a** showed the remarkable pharmacokinetic profile with maximum plasma concertation, when it was administered orally. Compound **87a** was found to be moderately active *in vivo*. Further modification in the structure of **87a** is required for the development of an effective molecule for the treatment of tuberculosis (Zhao et al., 2020).

### Formyl Peptide Receptor 1 (FPR1) Antagonist Activity

Inspired by a significant role of formyl peptide receptors in the regulation of inflammatory reactions in various diseases (Migeotte et al., 2006), 96 chromene analogs were tested to their specific FPR-1 antagonist activity and also reported inhibition of *f* MLF-induced intracellular Ca^2+^ mobilization in FPR1-HL60 cells. Compound **88** was found to be the most potent antagonist practically for FPR1- receptor (binding affinity Ki 100 nM) (Schepetkin et al., 2014).

### Miscellaneous Biological Activities

New amino-acid-containing conjugates of chromene-3-imidazoles, corresponding to natural isoflavonoids were reported (Figure 7). The author further evaluated these compounds for their *in-vitro* aldehyde reductase 2 (ALR2) inhibitory activities. All compounds of this series showed considerable activity with IC_50_ values 0.03 ± 0.08 to 4.2 ± 0.55 μM. Compound **89** showed the best inhibitory activity with a highly selective index against ALR1, which reduced blood glucose concentration and showed the development of cataract in a dose-dependent way (Gopinath et al., 2016).

New azo-group-containing 3-acetyl-2H-chromenes and 2H-chromene-3-carboxylate were prepared using the Knoevenagal condensation in the presence of active methylene with aromatic 5-arylazosalicylaldehydes. All these compounds were evaluated for their antioxidant activities by using hydroxyl, DPPH (2,2-diphenyl-1-picryl-hydrazyl-hydrate), and ABTS free-radical scavenging methods. Compound **90** substituted with the OCH_3_ group in the aryl azo molecule, showing the radical scavenging activity in the DPPH assay because e^−^ donating capability of –OCH_3_ group initiates phenyl ring activation. In addition, compound **91** showed superior hydroxyl scavenging activity, while compound **92** possessed a greater ABTS radical scavenging activity (Sivaguru et al., 2016).

## Structure-Activity Relationship (SAR) of 2H/4H-Chromenes

2H/4H-ch derivatives possess a wide-ranging spectrum of biological activities and are used extensively in medicine. The privileged literature shows that these heterocyclic rings had been broadly explored by modifying their structure in several ways either by coupling the 2H/4H-Ch ring with other heterocyclic scaffolds or by introducing diverse functional groups at different positions and evaluating their desired pharmacological activities. These structural conformational changes have discussed.

**Figure 8:**
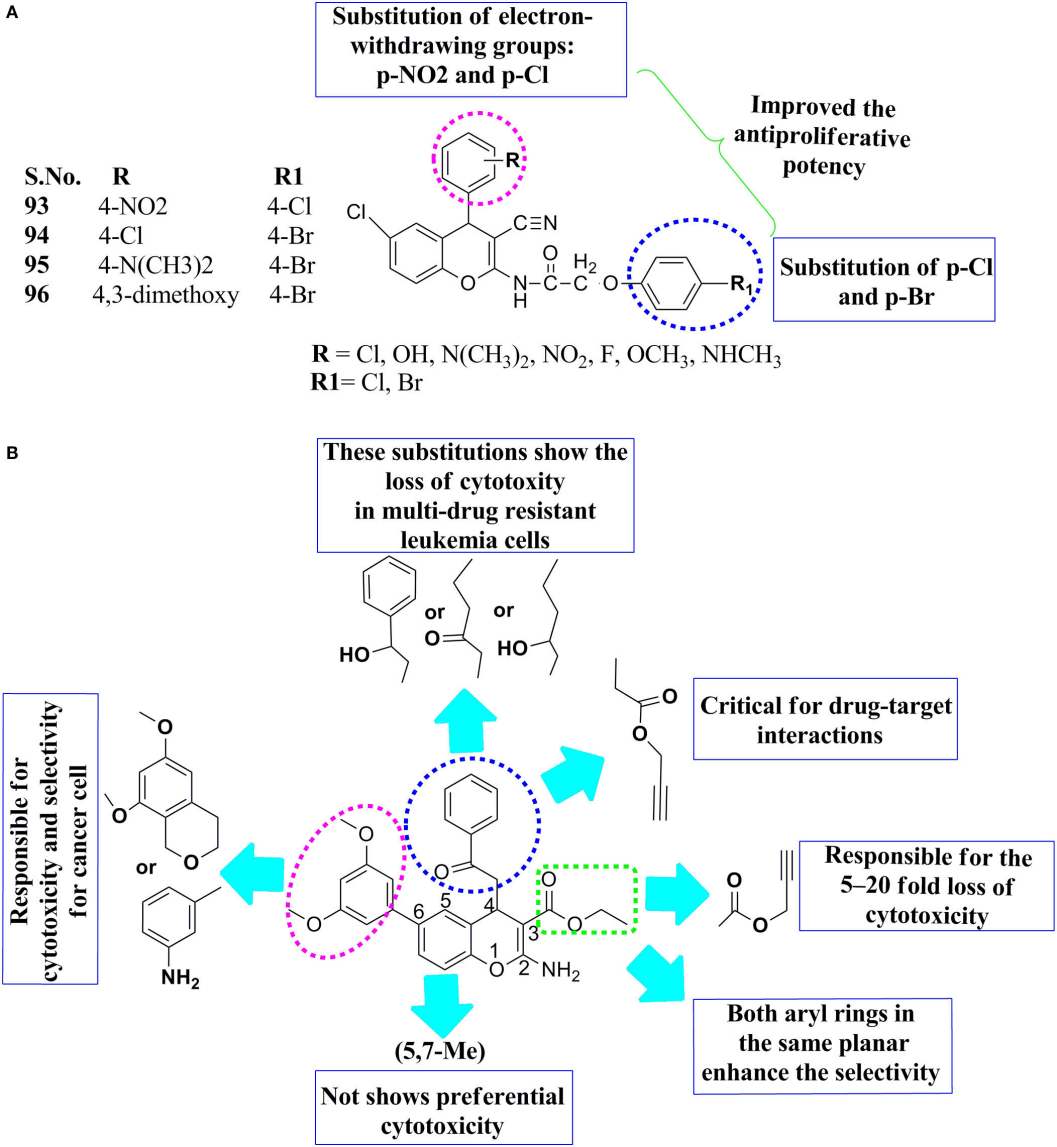
SAR features: **(A)** substituted N-(6-chloro-3-cyano-4-phenyl-4H-chromen-2-yl)-2-(4-chloro-phenoxy)-acetamides, **(B)** 4H-chromene-3-carboxylate analogs.

Substituted 4H-ch, SAR was reported that the substitution of p-nitro, p-Cl at the R position, and p-Cl and 4-Br R1 position on the basic backbone of the 4H-ch ring showed the effectiveness of compounds **93** and **94** toward anticancer activity (Figure 8A). Reasonable potency toward HT-29 human cancer cells was observed when the substitution was changed by the replacement of p-nitro with p-Cl or p- methoxy or p-amino-methyl at the R position and an electron-withdrawing group of p-Cl at R1. Compounds **95** and **96** had moderate anticancer effects because they contained 4-N(CH_3_)_2_,4,3-dimethoxy substitutions at the R position of the parent molecule and with p-Bromo substitution at the R_1_ position. The introduction of the electron-withdrawing (p-Cl) at the position R, while para-substitution electron-withdrawing (p-Br) at the R1 position had a promising anticancer effect. On the other hand, the substitution of –OH as a hydrophilic group at the R position and an e^−^-withdrawing substituent at the position R_1_ resulted in enhanced the anticancer activity. Consequently, the substitution of electron-withdrawing groups, for example, p-NO_2_ and p-Cl at R and p-Cl and p-Br at the R1 position, led to an improvement in the antiproliferative potency. This means that Br and -Cl atoms have a larger size and lower lipophilicity, which helps cause stronger interactions with the anticancer targets (Figure 8A) (Chauhan et al., 2020).

The SAR of the 4H ch derivatives was reported with their cytotoxic effect after some structural modifications. According to the prediction of SAR, the substitution of propargyl at both the third and fourth positions on the main motif of 4H-ch was important, a 5- to 20-fold loss of cytotoxicity was observed when the propargyl group was replaced with either ethyl or a propyl group (Figure 8B). The substituted aryl group of the 3′, 4′, and 5′ of the sixth structure positions of chromene can only accept minor substitutions, such as methylamino or methoxy groups. Inspired by these above SAR, features of chromene helps to optimize and improve the drug properties of chromenes, which may assist in future translational improvement. Besides, conformational flexible substitution at the sixth position of the 4H-ch, while ester group at the fourth positions were significant for its cytotoxicity concerning cellular selectivity and cytotoxicity against a multi-drug cancer-resistant cell line, namely as HL60/MX2. Even novel analogs were safe compared to the previous lead molecule, afford more support that the 4H-ch had a potential core motif to the development effective drug molecule against drug-resistant cancer therapy and delivered further evidence for future drug optimization (Puppala et al., 2016).

**Figure 9:**
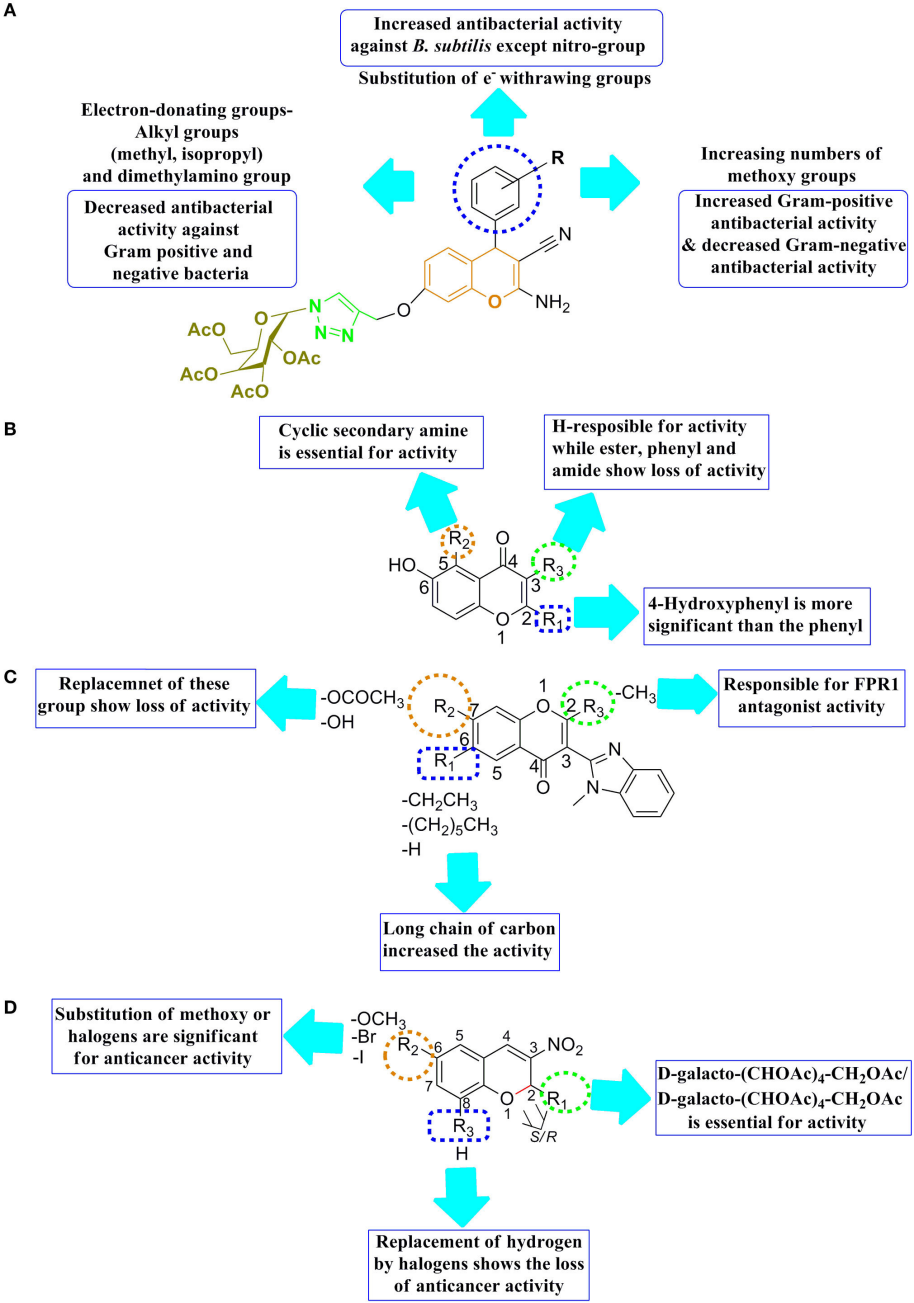
SAR features **(A)** 1H-1,2,3-triazole containing tethered 4H-chromene-D-glucose conjugates, **(B)** 4H-chromen-4-one analogs, **(C)** 3-(Benzimidazol-2-yl)-4H-chromen-4-one analogs, and **(D)** 2-glyco-3-nitro-2H-ch.

The SAR features of a series of 1H-1,2,3-triazole containing tethered 4H-chromene-D-glucose conjugates by the alteration of the group at R position were reported with *in-vitro* potency against the microorganisms such as *B. subtilis, S. aureus*, and *S. epidermidis* (Figure 9A). The SAR result revealed the following significant point, such as (**1**) substitution with an electron-withdrawing group at the position of R in benzene increased the potency against the Gram-positive such as *B. subtilis* except for a nitro group; (**2**) substitution with an electron-donating group at the R position, such as alkyl (isopropyl, methyl) and dimethylamino group, reduced the microbial potency toward Gram-positive/negative bacteria; and (**3**) increasing the number of methoxy groups at the R position resulted in decreased Gram-negative antibacterial activity and increased Gram-positive antibacterial activity (Thanh et al., 2019).

SAR features of 4H-chromen-4-one analogs were reported by Zhao et al. (2020) for the antituberculosis activity that the second, third, and fifth positions of chromene motif were important for antituberculosis activity. Therefore, 4-hydroxyphenyl was more important than the unsubstituted phenyl at the second position, resulting in improving antituberculosis activity (Figure 9B). While the substitution of hydrogen group at third position exhibited the antituberculosis activity, the replacement of hydrogen with other substitution showed reduction in antituberculosis activity. Importantly, the introduction of cyclic second amine at the fifth position was significant for antituberculosis activity and helps to increase the antagonist potency of chromenes (Zhao et al., 2020).

The SAR of 3-(Benzimidazol-2-yl)-4H-chromen-4-one analogs was reported that the second and seventh positions of chromene motif are important; therefore, CH_3_/CF_3_ (small hydrophobic) at the second position, resulting in showing better EPR1 inhibitory activity (Figure 9C). The seventh position tolerated a wide-range modification and incorporated for improving EPR1 antagonist potency of chromenes (Schepetkin et al., 2014).

A series of new 2-glyco-3-nitro-2H-chromenes for anticancer activity were reported by Luque-Agudo et al. (2019). The SAR of these series of compounds were constructed that 2H ch was an important motif for the development of anticancer lead molecule (Figure 9D). The racemization at the second position either the substitution of d-galacto-(CHOAc)_4_-CH_2_OAc or d-galacto-(CHOAc)_4_-CH_2_OAc, showed considerable anticancer activity. The sixth position of chromene was also important for a wide range of substitution likely methoxy and halogens, improving to anticancer potency of molecules. On the other hand, replacing hydrogen at the eighth position exhibited loss of anticancer activity (Luque-Agudo et al., 2019).

## Conclusion

Chromenes are an important class of heterocyclic compounds with several promising biological profiles, including anticancer, antimicrobial, antihyperglycemic, anticonvulsant, antitubercular, EPR1 antagonist, MAO inhibitor, and anticholinesterase. In particular, the 2H/4H-ch ring has been used as a template for designing new scaffolds, which have potential use in antitumor molecule discovery and other biological activities. Several methods of synthesis for the preparation of a 2H/4H-ch ring and its derivatives are available, such as microwave irradiation, one-pot synthesis, metal catalyst assisted, solvent-free synthesis, and other environmentally friendly methods. Knoevenagel condensation, aldol condensation, and Michael addition synthetic strategies are most commonly employed for the synthesis of 4H-ch analogs because of the ready availability of starting materials and as they produce a higher yield of product through one-pot multicomponent synthesis, while Witting-Horner-Emmons and Suzuki-Miyaura cross-coupling pallado- or rhodium-catalyzed synthesis are used for 2H-ch analogs. The various designing strategies and SAR studies of 2H/4H-ch are represented in this current review, demonstrating the useful structure features for medicinal chemists looking for appropriate substitutions by designing the effective chromene molecules of interest. According to the summary of SAR, a wide variety of C3-ester substitution and C4 benzopyran with modified arty ether at chromenes are more favorable for the antagonist activity toward the ER, particularly for ERα in cancer. Importantly, substituted fourth position with lipophilic electron-withdrawing of 4H-benzo[h]chromenes are incorporated with halogen-substituted benzene, produce significant anticancer activity and selectivity toward c-Src kinase inhibition. Besides, the halogen or methoxy group at the C-6 position of the 2H-ch scaffold is essential for the anticancer activity. The modifications at the C-4 and C-6 positions of chromenes are crucial for inducing the effectiveness by the inhibition of Sarco/endoplasmic reticulum calcium-ATPase (SERCA) through a distinctive binding interface and altering the SERCA stimulation in cells. Additionally, the CN group at the third position and aromatic substitution at the fourth position of chromene motif as well as lipophilic functional group at the sixth position of chromene are important for anticancer activity, enhancing to the cell apoptosis via activation of caspases and arresting the cell cycle phase. Similar substitution patterns of chromenes are reported for potent antimicrobial activities in privileged literature, but the underlying mechanism of these analogs to inhibit microorganism remains unexplored; therefore, it needs to be further investigated. These structural modifications are also helpful for rational design and the development of several chromene molecules for various targeted biological activities in a diverse way.

## Future Perspective

The effectiveness and potency of chromene analogs toward various tumor cells showed that these molecules should be established as a significant drug candidate for the treatment of various cancers. Chromenes have the potency for inhibiting the polymerization of tubulin by the binding at or nearby to the colchicine binding pocket in the cancer cell and has enormous significance in tumor research. Nevertheless, the experimental effectiveness of tubulin inhibitors is now obstructed by drug-mediated resistance through the overexpression of the efflux pumps of the membrane, that is, the permeability glycoprotein (Pgp). Pgp is a member of a superior family of ABC proteins and is encoded by the MDR1 gene in humans. A decrease in the intracellular concentration of transported substrates mediated *via* MDR proteins is the foremost factor in the failure of cancer chemotherapy. Despite this, chromenes have the potency to inhibit the SERCA and alter its expression in cancer cells, resulting to improve effectiveness for the treatment of multi-drug-resistant cancer cells. Due to all above these potential activities of chromenes, they have opened new endeavor for the discovery and development of potent chromenes drug molecules for desire biological activities. Even various chromenes have been reported with biological activities, there remains probably a gap for the perfection of efficacy and selectivity. This may be effectively achieved *via* (a) modification or ring fusion of chromene with other heterocyclic motifs, (b) generating stereospecific center in chromene by stereoselective synthesis, (c) by investigating the gene expression effect of chromenes in cancer or microbial studies, and (d) conjugated with desired metals or carbohydrates for triggering cell apoptosis due to limited accumulation in cells. Another problem with chromenes is limited solubility in the aqueous phase, which restricts their clinical development. For the enhancement of solubility, chromenes may be substituted either hydrophobic groups or incorporated in the form of a prodrug. Moreover, co-crystallization, nanoparticle, or other pharmaceutical doses may also be helpful to enhance the solubility of chromenes. Hence, drug research in the future should continue to discover newly chromene-containing potent antitumor drug candidates with dual molecular mechanisms, which would attract immense attention in drug discovery as well as afford new therapeutic choices for the cancer treatment.

## Author Contributions

VR and JL designed the study and wrote the manuscript. All authors read and approved the final manuscript.

## Conflict of Interest

The authors declare that the research was conducted in the absence of any commercial or financial relationships that could be construed as a potential conflict of interest.

## Abbreviations

IC_50_: 50% growth inhibition
SAR: Structure-activity relationship
3D-QSAR: Three-dimensional quantitative structure-activity relationships
MgO: Magnesium oxide
PoPINO: Potassium phthalimide-N-oxyl
DBU: 1,8-Diazabicyclo[5.4.0]undec-7-ene
MTT: 3-(4, 5-Dimethylthiazol-2-yl)-2,5-diphenyl tetrazolium bromide
ER: Estrogen receptor
MES: Maximal electroshock induced seizure tests
scPTZ: Subcutaneous pentylenetetrazol.
